# Emerging MR Imaging and Spectroscopic Methods to Study Brain Tumor Metabolism

**DOI:** 10.3389/fneur.2022.789355

**Published:** 2022-03-16

**Authors:** Manoj Kumar, Ravi Prakash Reddy Nanga, Gaurav Verma, Neil Wilson, Jean Christophe Brisset, Kavindra Nath, Sanjeev Chawla

**Affiliations:** ^1^Department of Neuroimaging and Interventional Radiology, National Institute of Mental Health and Neurosciences, Bengaluru, India; ^2^Department of Radiology, Perelman School of Medicine at the University of Pennsylvania, Philadelphia, PA, United States; ^3^Department of Radiology, Icahn School of Medicine at Mount Sinai, New York, NY, United States; ^4^Median Technologies, Valbonne, France

**Keywords:** brain tumor, chemical exchange saturation transfer (CEST), MRI and MRS, glioblastomas (GBMs), neurometabolites, two-dimensional correlation spectroscopy (2D-COSY), three-dimensional echo-planar spectroscopic imaging (3D-EPSI)

## Abstract

Proton magnetic resonance spectroscopy (^1^H-MRS) provides a non-invasive biochemical profile of brain tumors. The conventional ^1^H-MRS methods present a few challenges mainly related to limited spatial coverage and low spatial and spectral resolutions. In the recent past, the advent and development of more sophisticated metabolic imaging and spectroscopic sequences have revolutionized the field of neuro-oncologic metabolomics. In this review article, we will briefly describe the scientific premises of three-dimensional echoplanar spectroscopic imaging (3D-EPSI), two-dimensional correlation spectroscopy (2D-COSY), and chemical exchange saturation technique (CEST) MRI techniques. Several published studies have shown how these emerging techniques can significantly impact the management of patients with glioma by determining histologic grades, molecular profiles, planning treatment strategies, and assessing the therapeutic responses. The purpose of this review article is to summarize the potential clinical applications of these techniques in studying brain tumor metabolism.

## Introduction

Gliomas are the most common primary brain tumors of the central nervous system (CNS) in adults and carry a grim prognosis (1–3). Proton magnetic resonance spectroscopy (^1^H-MRS) is a valuable tool for the non-invasive assessment of metabolic alterations present within brain tumors (4). Several prior studies have reported potential utilities of ^1^H-MRS in evaluating histological grades, molecular profiles, determination of prognosis, and assessment of treatment response to established novel therapies in patients with gliomas (5–10).

Despite presenting promising findings in clinical practice, the widely used single-voxel or single-slice two-dimensional ^1^H-MRS methods suffer from some limitations. First, the conventional ^1^H-MRS methods are constrained by the incomplete sampling of gliomas due to limited coverage of a region of interest. Consequently, it may not always be possible to interrogate the entire tumor volume, and as such, metabolic alterations from the essential regions of glioma may not be sampled. Conversely, the sampled large voxels from solid/contrast-enhancing regions of gliomas might include the edges of necrotic regions, peritumoral edematous regions, normal brain tissues, or a combination of these tissue compartments. In such circumstances, metabolite levels in a particular tissue region of a tumor might be influenced by contributions from adjacent tissue compartments, thereby lowering the diagnostic accuracy of ^1^H-MRS by falsely including features that do not actually exist in that particular tumor region. Second, the neoplastic cells in high-grade gliomas have a propensity to infiltrate beyond contrast-enhancing regions in the normal brain parenchyma, often along the myelinated axons and blood vessels (11–13). However, standard ^1^H-MRS sequences do not often map the widespread metabolite abnormalities and suffer from the incomplete sampling of a glioma. Third, it is challenging to resolve several overlapping resonances existing over a small chemical shift range using conventional ^1^H-MRS techniques, hampering the reliable separation of several metabolites, which are of critical importance in understanding the metabolic processes occurring within these gliomas. Over the last several years, alternative MR-based techniques have been developed and successfully implemented in research and clinical settings to unravel the metabolic profiling of gliomas.

In this review article, we will provide an overview of emerging metabolic MRI techniques that have revolutionized the field of neuro-oncology in the recent past. These techniques include three-dimensional echoplanar spectroscopic imaging (3D-EPSI), two-dimensional correlation spectroscopy (2D-COSY), and chemical exchange saturation technique (CEST) MRI methods. Recently, ^13^C hyperpolarized and deuterium-based techniques have also gained momentum for studying metabolic pathways in brain tumors. While it is not in the scope of the current review for presenting a discussion on these novel multinuclear spectroscopic techniques, readers are referred to excellent articles available in the literature for a detailed overview on basic concepts and potential applications in neuro-oncology (14–20).

## Three-Dimensional Echoplanar Spectroscopic Imaging

Three-dimensional echo planar spectroscopic imaging (3D-EPSI) is an elegant method that provides high-resolution (nominal voxel size = 0.1 cm^3^) volumetric metabolite maps covering supratentorial and infratentorial brain regions in a clinically acceptable acquisition window (21, 22). These volumetric maps can be spatially co-registered with anatomical images to facilitate mapping of metabolite alterations from normal brain parenchyma and from different tumor regions (central core, solid/contrast-enhancing, and peritumoral regions) with minimal partial volume averaging, thus projecting a more comprehensive representation of a tumor true spatial extent (23). Moreover, the whole-brain data acquisition scheme obviates the subjectivity and user bias for placing voxels in a tissue of interest (24, 25).

On routine clinical MR systems (1.5 or 3 T), the whole brain 3D-EPSI data is acquired using a spin-echo sequence with parallel imaging scheme using generalized autocalibrating partially parallel acquisition (GRAPPA) (26). This sequence requires strong, fast switching gradients with excellent eddy current performance. It takes ~17–18 min to acquire a 3D-EPSI sequence that involves interleaved metabolite and water reference scans. The water scan is employed to improve the reconstruction and analysis of metabolite maps in the presence of magnetic field inhomogeneity and drift, besides using it as a reference signal for metabolite concentration scaling. To reduce lipid signal contamination from the skull and scalp, an inversion recovery prepared lipid inversion nulling is generally used in addition to employing an outer volume saturation band covering the skull base.

In neuro-oncology, multiple studies have shown the potential clinical utility of 3D-EPSI in studying brain tumor metabolism (23, 27–32). Glioblastomas (GBMs) are well known for their infiltrative nature with finger-like tentacles extending into the normal brain parenchyma like “mixing black and white sand” together, making differentiation of tumor cells from normal brain cells challenging on postcontrast T1-weighted images (33, 34). It is well established that cellular and metabolic alternations initiate much earlier than the appearance of actual lesions or anatomical changes in any pathological conditions, including brain tumors. By using 3D-EPSI, metabolite profile can be assessed both from the supratentorial and infratentorial brain regions simultaneously. Any deviation in the normal metabolite profile from normal-appearing brain tissues may be indicative of tumor infiltration. More importantly, these infiltrative tumor regions are generally associated with tumor recurrence and treatment failure (35, 36). Hence, it is crucial to develop a roadmap for precise delineation of tumor margins that will eventually aid in appropriate individualized therapeutic planning, including maximal safe tumor resection and delivery of accurate radiation therapy in these patients. To this end, 3D-EPSI derived total choline (tCho)/total N-acetyl aspartate (tNAA) maps were used in a study to detect regions of microscopic occult tumor infiltration beyond the areas of contrast enhancement in patients with GBM (26, 28, 37). The strong positive correlations were observed between tCho/tNAA ratios and quantitative measures of tumor infiltration (Sox2-positive cell density and *ex-vivo* fluorescence signals), supporting the notion that whole-brain metabolic maps can be used for reliable detection of infiltrative tumor regions (23).

Moreover, the investigators found that metabolically active disease does not always receive appropriate dose coverage in conventional radiation therapy planning in a routine clinical setting. The authors suggested using whole-brain tCho/tNAA maps in defining a target volume for delivering a maximum dose of radiation to active tumor regions for improved clinical outcomes in patients with GBM.

**Figure 1:**
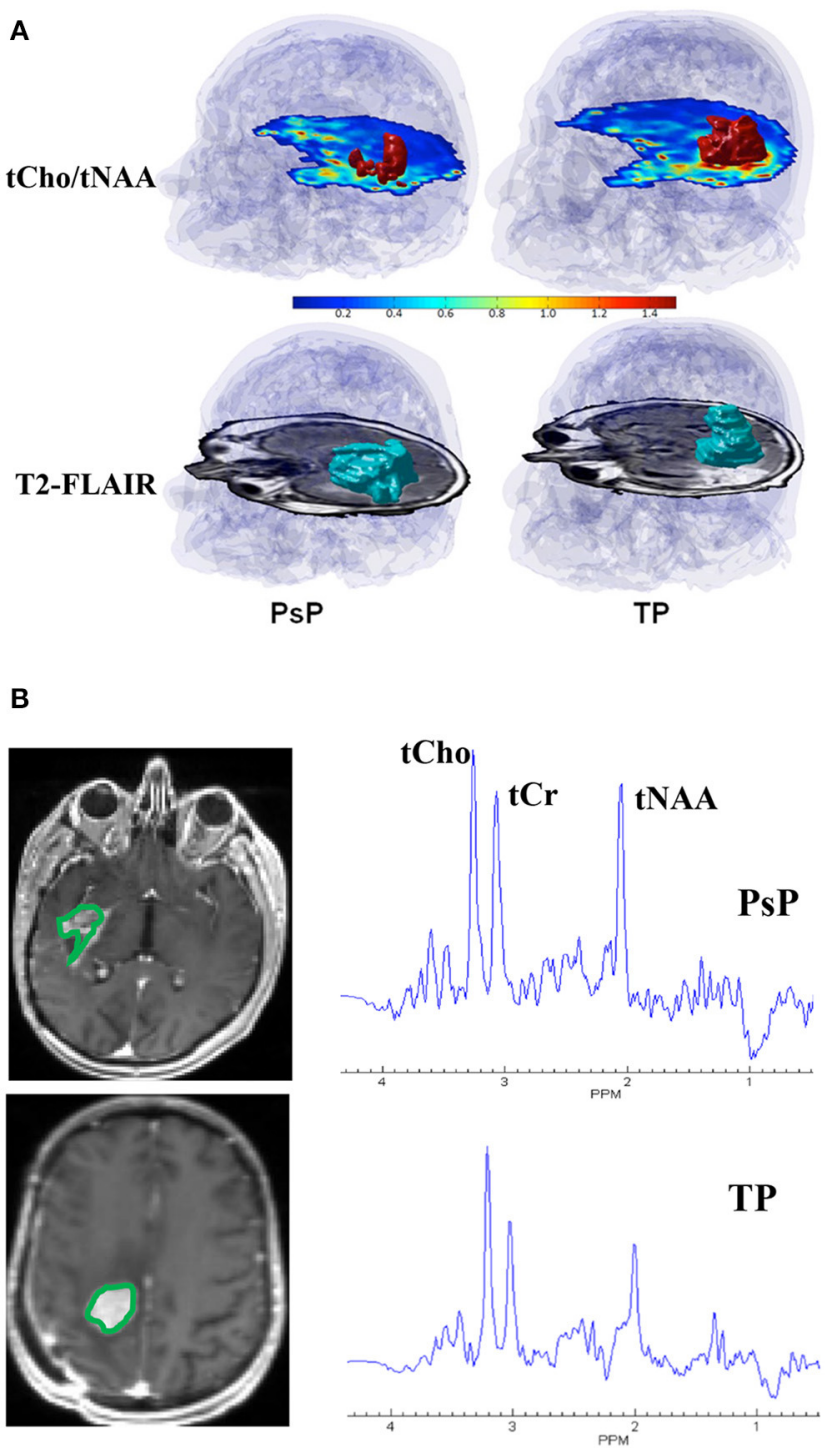
**(A)** Top Row: tCho/tNAA maps from patients with representative PsP (left) and TP (right) showing red color 3D volumes corresponding to voxels that exceed a threshold value of 0.85 for tCho/tNAA overlaid on the inferior most slices of tCho/tNAA maps encompassing the neoplasm. A transparent blue shows an outline of the brain and skull derived from a coregistered T_1_-weighted MPRAGE image. Bottom Row: T_2_-FLAIR image from these two patients showing blue color 3D volumes corresponding to FLAIR abnormality that is approximately similar in size (227 voxels, 24.4 cm^3^ for PsP and 219 voxels, and 23.6 cm^3^ for TP). Please note that the red 3D volume of tCho/tNAA is smaller in patients with PsP than patients with TP despite the presence of approximately comparable volumes of FLAIR abnormality, emphasizing the importance of 3D-EPSI in ascertaining the true extent of metabolic abnormalities in patients with TP and PsP. **(B)** Summed ^1^H-MRS spectra encompassing the entire volumes of contrast-enhancing regions of neoplasms from patients with TP and PsP are shown. Please note, higher tCho/tNAA ratio in TP than in PsP.

Surgical resection followed by concurrent chemoradiotherapy (CCRT) along with adjuvant temozolomide (TMZ) is the current standard of care treatment for patients with GBM (38). However, within 6 months after completing CCRT, a contrast-enhancing lesion within the radiation field at the site of original tumor or resection margins appears (39). While this lesion may represent true progression (TP) of a neoplasm, it may also reflect either a predominant treatment effect/pseudoprogression (PsP) that is mediated by TMZ-induced increased vascular permeability and inflammatory response. Specifically, PsP is a subacute and posttreatment reaction that subsequently regresses or remains stable (40). Patients with TP are considered for repeat surgery or for alternative treatment strategies (41). On the other hand, patients with PsP are associated with a favorable prognosis and these patients are usually continued on adjuvant TMZ therapy (42, 43). Therefore, it is essential to differentiate TP from PsP for making informed decisions on future therapeutic interventions and for prognostication of disease in these patients. Using 3D-EPSI, our group demonstrated the clinical utility of tCho/tNAA and tCho/total creatine (tCr) maps in distinguishing TP from PsP with a discriminatory accuracy of over 90% (27). We observed significantly higher tCho/tNAA from contrast-enhancing, immediate, and distal peritumoral regions of GBMs in TP than those in patients with PsP. In addition, significantly elevated tCho/tCr levels from the contrast-enhancing areas were observed in TP compared with PsP. When these parameters were incorporated into multivariate logistic regression analyses, a discriminatory model with a sensitivity of 94% and a specificity of 87% was observed in distinguishing TP from PsP (27). Representative 3D-EPSI derived volumes of tCho/tNAA, and T_2_-FLAIR abnormalities from patients with TP and PsP are demonstrated in Figure 1A. In addition, summed ^1^H-MRS spectra encompassing the entire volumes of contrast-enhancing regions of neoplasms from these two patients are given in Figure 1B.

In another study, our group documented the clinical utility of 3D-EPSI in assessing the deleterious effects of whole-brain radiation therapy on normal brain parenchyma in patients with metastases (32). This study reported a significant increase in tCho/tCr levels in the hippocampus and genu of the corpus callosum at a 1-month postirradiation period relative to the baseline MRI study. These alterations in tCho levels support the idea that radiation therapy disrupts the metabolism of the normal brain tissues secondary to the breakdown of the myelin sheath and cell membranes following radiation-induced damage to oligodendrocytes. In addition, trends toward decrease in tNAA/tCr ratio were observed from the hippocampus (32).

Numerous other studies have also reported the immense clinical potential of 3D-EPSI in characterizing histological grades of gliomas, mapping distribution of glycine in gliomas, defining target volumes for radiation therapy in GBMs, and evaluating treatment response to immunotherapy and electric field therapy in patients with GBM (23, 28–31). Collectively these studies have reported that volumetric EPSI sequence can evaluate the spatial extent of metabolic alterations with high accuracy, which is essential for assessing disease burden in patients with glioma.

Mutation in isocitrate dehydrogenase (IDH) 1 and 2 families of enzymes in the TCA cycle causes NADPH-dependent reduction of α-ketoglutarate to 2-hydroxyglutarate (2HG), resulting in a two-to-three order of magnitude increase in the cellular 2HG concentration, which otherwise is present in vanishingly small quantities in the normal human brain regions. This mutation occurs at a single amino acid residue of the IDH active site resulting in loss of the enzyme's ability to catalyze the conversion of isocitrate to α-ketoglutarate. Several studies have reported that patients with glioma harboring IDH mutation demonstrate a better response to chemoradiation therapy and prolonged overall survival outcomes than those harboring IDH wild-type alleles (44, 45), thus emphasizing the importance of non-invasive identification of IDH mutant gliomas. An alteration in tumor metabolism results in the aberrant accumulation of an oncometabolite 2HG, which has been considered as a putative marker for identifying IDH mutant gliomas (10, 45). Structurally, the oncometabolite 2HG consists of a 5-spin system and the scalar (J) coupling pattern of 2HG leads to several multiplets with spectral peaks centered around 4.02 (H2), 1.9 (H3 and H3′), and 2.25 ppm (H4 and H4′) spectral locations. The noninvasive detection of 2HG on conventional ^1^H-MRS is challenging due to the extensive overlap of its resonances with those from metabolites, namely, NAA, glutamate (Glu), glutamine (Gln), gamma-aminobutyric acid (GABA), and lipids. Some previous ^1^H-MRS studies have employed sophisticated acquisition and postprocessing strategies for reliable *in-vivo* detection and quantification of 2HG (46–48). It is well known that improved spectral resolution and reduced overlap can be achieved by applying a longer echo time (TE) to a standard point resolved spectroscopy (PRESS) sequence in order to take advantage of the differences in J-evolution observed in the coupled spin system (49). In a study, Choi et al. conducted several quantum mechanical simulations and finally used an optimal TE of 97 ms for the detection of 2HG signals from gliomas scanned on a 3 T MR system. The authors reported 100% sensitivity and specificity for detecting 2HG in gliomas (47). In a related study, the investigators used a triple refocusing sequence with a TE of 137 ms for improved detection of 2HG by successfully suppressing the confounding signals from Glu, Gln, and GABA (48). Using an innovative approach in a recent study, new lipid basis sets were used for detecting 2HG signals especially in those brain tumors, which exhibited high lipid resonances. Despite presenting encouraging findings, all these studies employed single voxel and/or single slice multivoxel ^1^H-MRS methods. Lately, a high-resolution whole-brain ^1^H-MRS technique has been proposed to map the oncometabolite 2HG from the entire volume of IDH mutant gliomas (50). Another recent study has demonstrated significantly higher tCho/tCr and tCho/tNAA ratios in IDH mutant than in IDH wild-type gliomas using whole-brain ^1^H-MRS (51). Taken together, this wide range of clinical applications provide a strong impetus to use the 3D-EPSI sequence for the non-invasive quantification and assessment of metabolite alterations in gliomas.

The volumetric EPSI sequence is also associated with some limitations that include the effects of magnetic field inhomogeneity, especially from frontal and brain stem regions that may limit the spectroscopic characterization of tumors located in those particular regions. To avoid the inclusion of low-quality spectroscopic data, an automatic quality assurance procedure is used for each voxel using several metrics such as Cramer-Rao lower bounds (CRLB), line shapes, CSF partial volume contribution, and degree of residual water and lipid signals. The voxels that do not meet these predefined criteria are excluded from the final data analysis (21). Given that the volumetric EPSI sequence provides reliable metabolic information from different regions of gliomas and multiple normal brain regions simultaneously, we expect this sequence to be used widely in neuro-oncological applications in the near future. Moreover, the implementation of the whole-brain 3D-EPSI sequence on 7 T MRI scanners will open more avenues for studying brain tumor metabolism.

## Two-Dimensional-Correlation Spectroscopy

**Figure 2:**
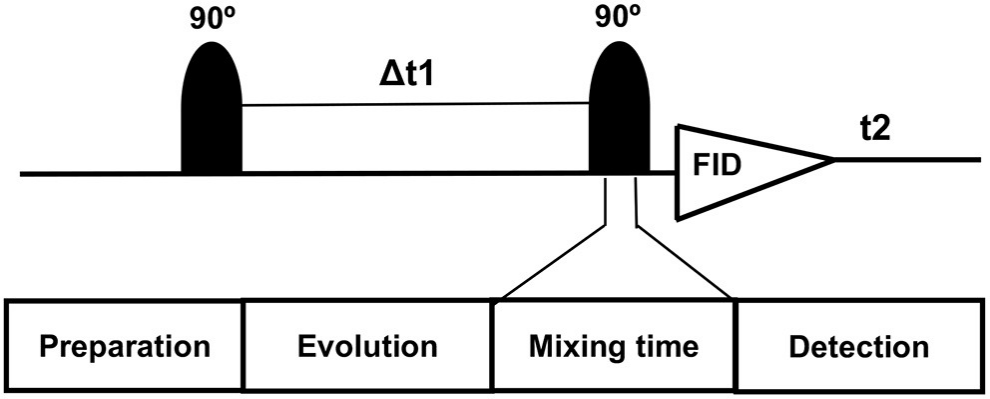
Basic pulse diagram of a 2D-NMR experiment preparation time allows the nuclei in the sample to reach equilibrium with the static external magnetic field environment and solvent suppression is performed. During the evolution period, the magnetization of spins evolves. This time domain is incremented during the 2D-NMR experiment. During mixing time, coherence transfer takes place between J-coupled spins. During the detection period, transverse magnetization is recorded.

**Figure 3:**
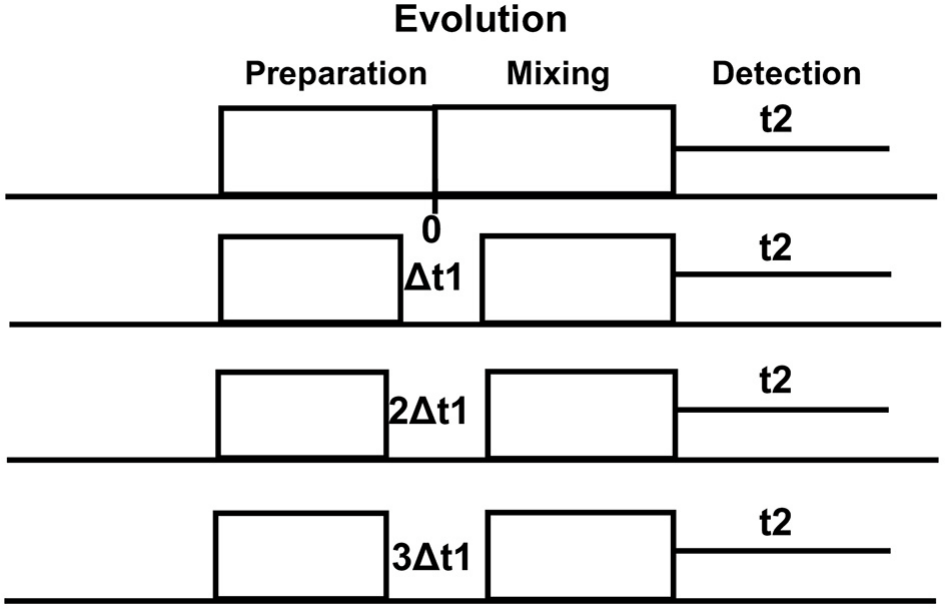
Multiple (~64) evolutionary periods (Δt1 increments) are collected in a typical 2D-NMR experiment which is a simple series of 1D-NMR experiments acquired with different timings. Higher resolution in t2 (direct dimension) costs little time, but the higher resolution in t1 (indirect dimension) adds directly to the acquisition time of the experiment.

On conventional one-dimensional (1D) ^1^H-MRS, spectral peaks due to methyl, methylene, and methane protons from the number of metabolites, namely, NAA, N-acetyl aspartyl glutamate (NAAG), Glu, Gln, GABA, and 2HG neurometabolic peaks severely overlap in the spectral region of 2–4 ppm, often confounding the detection and quantification of metabolite concentrations. In contrast, the 2D-COSY method offers the ability to identify potentially overlapping resonances of metabolites by dispersing the multiplet structure of scalar (J)-coupled spin systems into a second spectral dimension and by exploiting the unlikely possibility that two metabolites would share identical chemical shifts in two dimensions (52–55). A basic pulse sequence of a 2D-COSY consists of preparation time that allows the nuclei in the sample to reach equilibrium with the static external magnetic field environment and during which water suppression is performed. During the evolution period, the magnetization of spins evolves. This time domain is incremented during a pulse sequence. During mixing time, coherence transfer occurs between scalar (J)-coupled spins, and finally, transverse magnetization is recorded during the detection period (Figure 2). In a 2D-COSY experiment, the MR signal is recorded as a function of two-time variables, t_1_ (incremented time delay) and t_2_ (fixed time delay) (Figure 3). The series of 1D-free induction decays are Fourier transformed along the first dimension in t_2_ followed by another Fourier transformation in t_1_. The signals of each transformation may differ in amplitude and/or phase. Higher resolution in t_2_ (direct dimension) costs little time, but the higher resolution in t_1_ (indirect dimension) adds directly to the acquisition time of the experiment.

**Figure 4:**
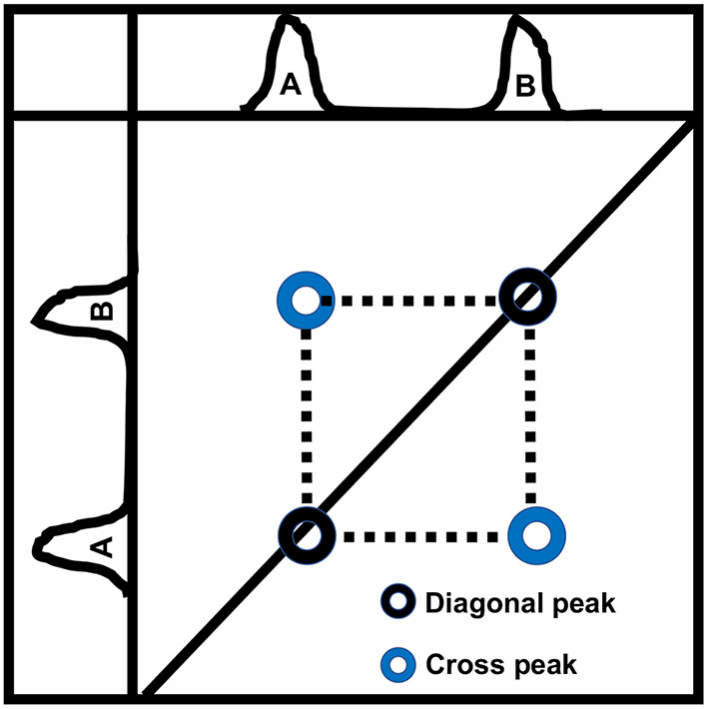
A schematic diagram showing a typical 2D-COSY spectrum. Two types of peaks are observed. Auto peak/diagonal peak indicates that protons from a chemical moiety are not J-coupled with protons of a neighboring moiety. Cross-peak/off-diagonal peak indicates spin-spin couplings between two protons up to three bonds apart (vicinal coupling).

As shown in Figure 4, a typical 2D-COSY spectrum comprises of two types of peaks (a) diagonal peak, which indicates that protons are not J-coupled with neighboring protons and (b) cross-peak or off-diagonal peak, which indicates spin-spin couplings between two protons up to three bonds apart (vicinal coupling). Though well-separated, the cross-peaks show lower signal intensity than primary resonances because they originate only from the small fraction of spins undergoing coherence transfer during t_1_ evolution.

Several earlier studies have reported the feasibility and clinical importance of *in-vivo* 2D-COSY sequence with human brains scanned at routine clinical scanners, mainly at 1.5 and 3 T MR systems (56–58). However, the introduction of ultra-high field (7T) MR systems has improved the sensitivity of 2D-COSY technique in detecting metabolites, particularly with overlapping resonance peaks. Previously, our group has implemented a 2D-COSY pulse sequence on a 7 T MR system and demonstrated enhanced signal-to-noise ratio (SNR) and increased chemical shift dispersion compared to a similar 2D-COSY scheme and voxel size acquired at a lower field strength of 3 T (59). In this reproducibility study, test-retest experiments of brain phantom using 2D-COSY sequence on 7 T revealed coefficients of variation (CVs) of 3–20% for most metabolites, suggesting high diagnostic accuracy of this method. A direct comparison of SNR indicated that most metabolite peaks were 2–3 times more intense at 7 T than at 3 T.

Furthermore, *in vivo* 2D-COSY spectra allowed the detection of several metabolite resonances from multiple brain regions of normal volunteers, and intersubject experiments revealed CVs of 4–26% for most metabolites (59). Collectively, these results demonstrate the feasibility, reliability, and clinical potential of the 2D-COSY sequence in unambiguously detecting additional metabolites in the human brain than what can be generally ascertained either by 1D studies at 7 T or analogous 2D-COSY studies at lower field strengths (1.5 or 3 T). Moving forward, our group implemented a non-uniformly weighted sampling (NUWS) scheme for faster acquisition of 2D-COSY data on a 7T MR system. The NUWS 2D-COSY facilitated a 25% shorter acquisition time while maintaining almost similar SNR in humans (+0.3%) and phantom experiments (+6.0%) to that of the uniform averaging method (60). This new approach could make the clinical applications of 2D-COSY sequences faster, easier, and more versatile.

In brain tumors, an elevated resonance of tCho at 3.2 ppm is generally observed on conventional ^1^H-MRS, indicating increased cellular proliferation (4, 7, 8, 61). This prominent resonance of tCho is composed of signals from free Cho, phosphocholine (PC), and glycerophosphocholine (GPC) (62). To fully understand the dysregulated phospholipid metabolism in brain tumors, it is essential to detect and quantify relative levels of PC and GPC (4). In a previous *in-vitro* ^1^H-MRS study from tumor extracts, it was found that PC was a predominant contributor to the tCho peak in high-grade gliomas (61). At the same time, GPC dominated in low-grade gliomas suggesting the potential role of relative amounts of PC and GPC in predicting tumor grades. The higher PC observed in high-grade gliomas has been attributed to elevated expression of choline transporters and/or enzymatic activities of choline kinase, and phospholipase-C (63). Furthermore, alterations in the PC/GPC ratio have been proposed as a marker of malignant transformation and treatment response (61). On conventional ^1^H-MRS, the individual peak components of tCho (free Cho, PC, and GPC) cannot be reliably resolved (4, 61, 64).

There has been an increasing interest in investigating the crucial roles of neurotransmitters in dynamic remodeling and regulation of metabolic pathways in brain tumors. Hence, it is imperative to understand the alterations in Glu, Gln, and GABA cycle as Glu is involved in the cellular anabolic pathways and is also associated with facilitating tumor invasion (65–67). Consequently, reliable detection of these metabolites is valuable for studying tumor metabolism. Detection of elevated lactate (Lac) in brain tumors may reflect elevated tumor glycolysis and/or poor tissue perfusion (68). However, reliable detection of Lac on 1D ^1^H-MRS is problematic due to intense co-resonant lipids signals that are also known to be present at elevated levels, especially in high-grade gliomas.

In a study, we published our initial experience of using 2D-COSY sequence on a 7 T MRI scanner for successfully identifying IDH mutant gliomas by unambiguously detecting resonances of 2HG besides reporting other clinically relevant metabolites (69).

**Figure 5:**
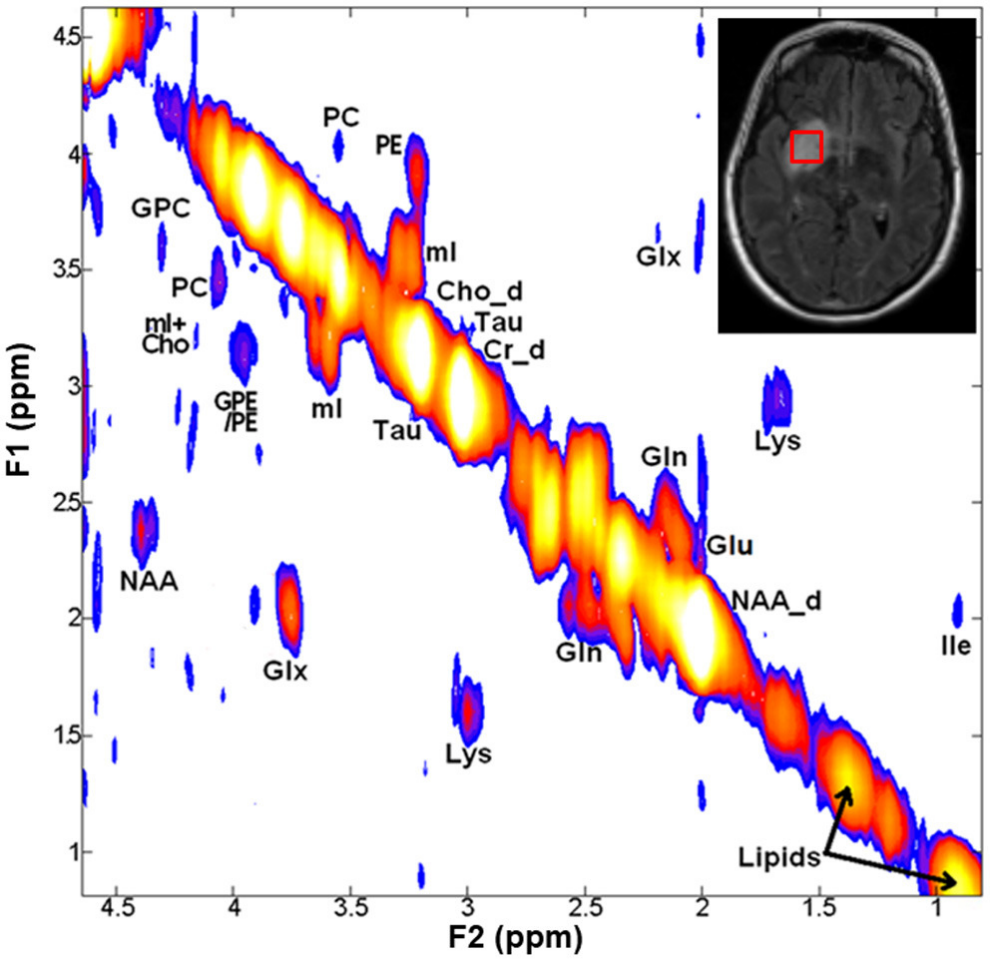
A 2D-COSY spectrum (from a voxel placed on hyperintense signal abnormality as visible on T2-FLAIR image, right inset) from a patient with grade-III astrocytoma harboring IDH wild-type genotype showing various metabolites. Abbreviations: choline (Cho); ethanolamine (Eth); glutamate (Glu); glutamine (Gln); glutamate + glutamine (Glx); glycerophosphocholine (GPC); isoleucine (Ile); lysine (Lys); Myo-inositol (mI); N-acetyl aspartate (NAA); phosphocholine (PC); phosphoethanolamine (PE); and taurine (Tau).

Representative 2D-COSY spectrum from a patient with grade-III astrocytoma harboring IDH wild-type genotype is shown in Figure 5. In another study, Ramadan et al. reported several characteristic metabolites from GBMs using 2D-COSY (70). Collectively, these studies suggest the potential utility of 2D-COSY in the characterization of brain tumors. However, future studies with larger patient populations are required to validate these encouraging findings.

We recognize the limitations of the 2D-COSY technique in terms of its relatively longer acquisition time (~17 min). However, future modifications of this pulse sequence would benefit from implementing acceleration techniques like matched accumulation or sparse sampling to reduce overall scan time. Quantification of metabolic resonances detected on 2D-COSY could also be improved by implementing a prior-knowledge-based fitting approach analogous to linear combination (LC)-model fitting on 1D-MRS rather than using a peak integration method that is generally used. ProFit is one such 2D prior-knowledge-based fitting algorithm, and adopting this program could improve the reliable quantification of brain tumor metabolites (52, 71, 72). Future developments also include designing multivoxel-based 2D-COSY sequences using concentric circular echoplanar encoding or spiral encoding schemes for facilitating faster data with greater anatomical coverage and higher spatial resolution.

## Chemical Exchange Saturation Transfer

Chemical exchange saturation transfer (CEST) is a relatively novel metabolic imaging modality that allows the detection of specific exogenous and endogenous metabolites/molecules present at very low concentrations (73–75). In CEST imaging, exchangeable solute protons present on the chemical functional groups such as hydroxyls (–OH), amides (–CONH), and amines (–NH_2_) resonate at a frequency different from the bulk water protons (off-resonance frequencies for -OH, –CONH, and –NH_2_ protons are ~ 0.5–1.5, ~3.5, and 1.8–3.0 ppm, respectively). These labile protons are selectively saturated using radiofrequency (RF) irradiation centered at the resonance frequencies of these protons. This saturation is subsequently transferred to bulk water pool when solute protons exchange with water protons leading to a decrease in the water signal proportional to the concentration of solute molecules and exchange rate of labile protons. While the saturation pulse is being applied, this process continues to decrease the water magnetization. Simultaneously, longitudinal relaxation processes return the saturated proton spins to their thermal equilibrium state until the system reaches a steady-state condition or the saturation pulse is turned off. This reduction in the water signal can be imaged by modifying fast imaging pulse sequences. For the exchangeable solute protons of interest, two factors that play a critical role in obtaining the optimal contrast are (i) the applied power and (ii) the duration of the RF pulse.

The CEST imaging provides a powerful contrast for detecting endogenous mobile proteins/peptides and neurometabolites such as Glu and Cr, which play a crucial role in tumor development, tumor growth, and disease progression (76–79). Therefore, studying these macromolecules and metabolites using CEST imaging techniques may provide valuable insights for understanding the brain tumor microenvironment.

### Amide Proton Transfer

Amide proton transfer imaging is a CEST technique developed to detect and quantitatively visualize endogenous mobile peptides and proteins present within the biological tissues (76, 80). Amide protons resonate at around 8.2 ppm on the MR spectrum and hence have a chemical shift of 3.5 ppm downfield from the water signal. Due to the prolonged exchange rate (~30 s^−1^) of amide protons, it is possible to obtain nearly complete saturation using a low-power, long-duration saturation RF pulse (81). APT imaging can also be performed on 3 T clinical MRI scanners mainly due to the slow exchange rate of amide protons. As an APT signal is dependent on the variations in tissue pH values, APT imaging may be helpful in determining the tumor pH after performing appropriate calibration of the signals (82).

It is widely believed that active tumor cells express higher concentrations of mobile protein and peptide components (83, 84). Also, metabolically active tumors produce a higher amount of lactic acid in the extracellular tumor microenvironment (85). This decrease in tumor pH should generate lower APT contrast due to the slower exchange rate of amide protons because the chemical exchange of protons from the amide group to water is base-catalyzed. On the contrary, higher APT contrast is generally observed from tumors than from normal tissues. The plausible reason for this observation could be that higher peptides/protein contents present within the tumors might offset the pH-dependent lower APT signals (80).

Using orthotropic glioma models in rats, a preclinical study reported the potential utility of APT imaging in differentiating viable tumors from radiation necrosis. While actively growing tumor regions exhibited hyperintensities on APT images, necrotic regions showed hypointense to isointense signal intensities (86). In addition, the intensity of the APT signal was shown to decrease in irradiated tumors at 3-day and 6-day of posttreatment periods relative to baseline suggesting APT imaging may aid in evaluating treatment response to radiation therapy in brain tumors. Several clinical studies have also demonstrated the great potential of APT-weighted imaging in delineating malignant neoplastic infiltration from peritumoral vasogenic edema, differentiating histopathological grades, and discriminating high-grade gliomas from primary cerebral lymphomas (87–89). In addition, some studies have documented significantly higher APT signals in TP than those with PsP in patients with GBM. This difference may be attributed to the presence of lower concentrations of mobile cytosolic proteins and peptides in PsP secondary to reduced cellular density and disrupted cytoplasm than those present in TP (84, 87).

Amide proton transfer-weighted imaging has also been used to identify IDH mutation status in gliomas (90–93). Intriguingly, lower APT signal intensities have been observed in IDH-mutant (phenotypically indolent tumors with more favorable prognosis) than in IDH-wildtype (phenotypically aggressive tumors with poor prognosis) gliomas (94–96). APT imaging has also been used for identifying O^6^-methylguanine-DNA methyltransferase (MGMT) promoter methylation status in GBMs. In a study, significantly lower APT signal intensities were observed in MGMT methylated (a prognostic marker for more favorable prognosis) than in MGMT unmethylated GBMs (97). In summary, APT imaging is fast emerging as a novel molecular MRI technique in neuro-oncology that can be used for classifying brain tumor types, determining histological grades and molecular profiles of gliomas, and evaluating treatment response in patients with glioma.

### Glutamate-CEST

**Figure 6:**
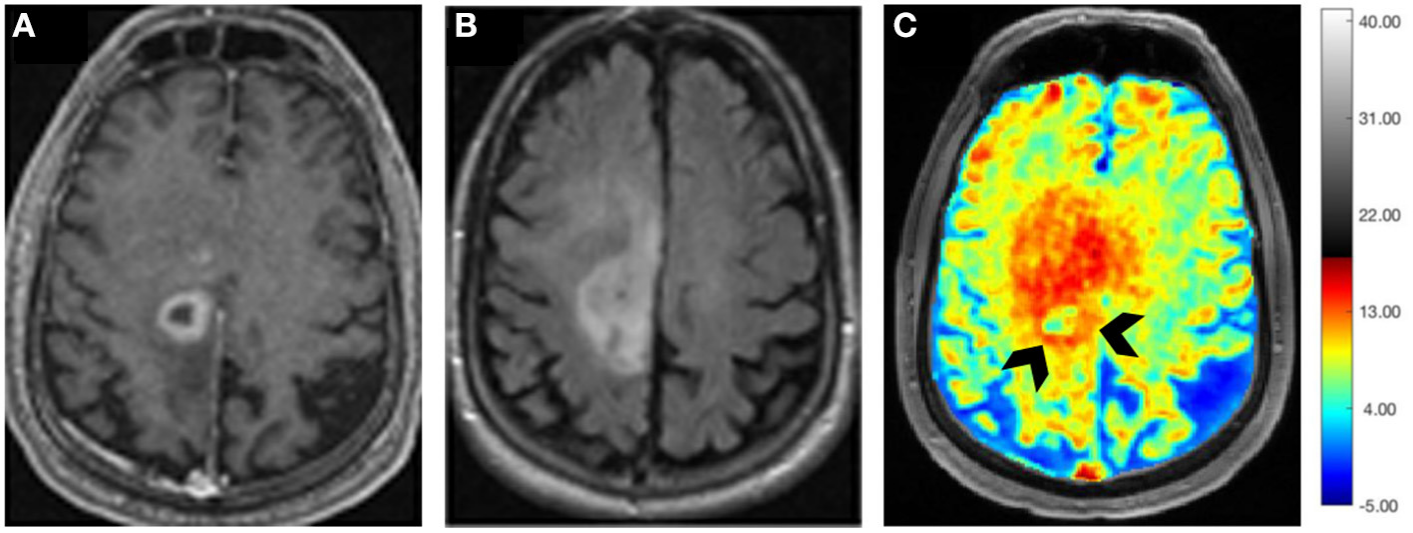
Axial postcontrast T_1_ weighted image **(A)** demonstrating a ring-enhancing GBM with hypointense vasogenic edema in the left frontal lobe, which is visible as a hyperintense mass on the corresponding T_2_-FLAIR image **(B)**. B_0_ and B_1_ field in-homogeneity corrected Glu-CEST map **(C)** showing high glutamate signals from contrast-enhancing ring (arrowheads) and the surrounding brain regions beyond the contrast enhancement, suggesting infiltrative nature of this neoplasm.

It has been demonstrated that Glutamate (Glu) exhibits a pH and concentration-dependent CEST effect between its amine protons observed at ~3.0 ppm downfield from bulk water protons. A CEST method for imaging Glu (Glu-CEST) can be utilized to generate high-resolution (in-plane resolution varies between 0.8 × 0.8 and 1.0 × 1.0 mm^2^) parametric maps for better understanding the role of this crucial metabolite in studying brain tumor metabolism (98–100). In a pilot study, higher Glu-CEST signals were observed from peritumoral regions of grade II–III gliomas compared to contralateral brain regions. Interestingly, these high Glu-CEST contrast regions were associated with seizure activities in these patients (101). These findings substantiate the notion that the peritumoral regions could be potential epileptogenic zones in gliomas. In an ongoing study at our institution, we are currently investigating the potential role of Glu-CEST imaging on a 7 T MR scanner for detecting occult neoplastic infiltration into the normal brain parenchyma and evaluating the extent of glutamatergic excitatory activity in patients with GBM (Figure 6). We believe that improved delineation of tumor margins (*precision diagnostics*) will aid in appropriate individualized therapeutic planning, including maximal safe tumor resection and enhanced delivery of radiation dose to actively proliferating regions of a GBM (*personalized therapeutics*), thus improving the quality of life and survival outcomes in these patients.

### Creatine-CEST

Creatine-CEST (Cr-CEST) provides an imaging method to evaluate free Cr non-invasively with much improved spatial and temporal resolution than standard ^1^H-MRS methods (102). Under normal physiological conditions, Cr-CEST contrast is observed predominantly from Cr with negligible contributions from phosphorylcreatine centered at +1.8 ppm downfield from water resonance. It has been shown that Cr-CEST imaging has over 1,000 times higher detection sensitivity compared to single-voxel ^1^H-MRS enabling high-resolution mapping of Cr signal within a biological tissue (103).

Using the 9L gliosarcoma rat brain tumor model, a previous study demonstrated lower Cr-CEST peak integral from tumor regions than from contralateral normal brain regions. Moreover, the Cr-CEST peak integral decreased further with tumor progression (79). Furthermore, significantly lower Cr-CEST peak integrals were found in F98 (more aggressive) rat brain tumor model than in the 9L tumors (less aggressive) (79). It is well known that F98 gliomas share the essential characteristics of human GBMs (Grade-IV gliomas, the most malignant brain tumors), such as invasiveness and formation of hypoxia and necrosis. On the other hand, 9L gliosarcoma grows slowly and homogeneously and these tumors rarely exhibit regions of necrosis (104–106). Taken together, the findings of Cr-CEST studies on rodent tumor models indicate that levels of Cr diminish as the tumor progresses and the degree of aggressiveness increases. These findings are in good agreement with previously published studies that have shown a decreased level of Cr and/or creatine kinase activity in several different types of cancers (107, 108).

Over the last decade, several encouraging findings have been obtained by using CEST imaging; however, these are mostly restricted to animal models or pilot clinical studies. To translate these promising results of CEST imaging into a routine clinical setting, some critical issues, including B_1_ inhomogeneities effects, especially in the temporal brain regions, the requirements for lower power deposition, longer scan time, and correct interpretation of CEST data need to be addressed. Sequences with radial or spiral k-space-filling strategies are being explored for CEST imaging to achieve a shorter acquisition time. Some developments such as parallel transmission, RF shimming, and post-B_1_ correction strategies are also currently in progress to address some of the shortcomings associated with CEST imaging.

## Summary

New advances in metabolic MRI and spectroscopic techniques have been evolving rapidly over the last few years. A comparative analysis of three metabolic techniques (3D-EPSI, 2D-COSY, and CEST imaging) is given in Table 1. These techniques will have significant roles in reshaping our understanding of brain tumor biology and metabolism for accurate tumor diagnosis, prognosis, and identification of new molecular targets for fostering the discovery of new treatments. However, it is critical to standardize and harmonize the acquisition parameters of these metabolic techniques for fast-tracking the translation and implementation into routine clinical workflows. Further progress in this field also requires data sharing and large multicentric, collaborative validation studies.

**Table 1:** Head-to-head comparison of three metabolic techniques (3D-EPSI, 2D-COSY, and CEST imaging).

**Type of characteristics**	**3D-EPSI**	**2D-COSY**	**CEST imaging**
General features	Simultaneous spatial-spectral encoding technique and potentially valuable tool for mapping metabolite profiles from multiple brain regions	Identify potentially overlapping resonances of metabolites by spreading out the multiplet structure of scalar (J)-coupled spin systems into a second spectral dimension and by exploiting the unlikely possibility that two metabolites would share identical chemical shifts in two-dimensions	Generates unique image contrast between tissues by targeting labile protons present within the endogenous molecules or exogenous compounds
Potential strengths	Offers advantages of greater spatial coverage and higher spatial resolution compared to multivoxel spectroscopic imaging techniques	Provides better dispersion of J-coupled peaks and unambiguously disentangles peaks present at similar chemical shift positions	Provides higher spatial resolution images for detecting metabolites with higher sensitivity compared to routine spectroscopy methods
Potential challenges	Longer shimming and acquisition times, the trade-off between spatial resolution and spectral bandwidth, prone to artifacts due to B_0_ inhomogeneity and magnetic field susceptibility	Long acquisition times to adequately sample t1 dimension, limited spatial coverage (typical single voxel size of 4–8cm^3^), and limited availability of post-processing tools	High RF power deposition, the requirement of ultrahigh magnetic field strength (≥7 T) for observing some fast-exchanging metabolites, e.g., glutamate, sensitivity to B_0_ and B_1_ field inhomogeneities
Future directions	Implementation of robust, whole-brain 3D-EPSI sequence on 7T MRI scanners	Development of sequences for allowing faster data acquisition with greater anatomical coverage and higher spatial resolution	Development of reliable multi-slice CEST imaging sequences
Potential clinical applications in brain tumors	Characterization of multiple brain tumor lesions, intratumoral heterogeneity, tumor infiltration, harmful effects of radiation therapy from multiple normal brain regions	Detection and quantification of metabolites whose resonances severely overlap with those of other metabolites. For example, separation of lactate from lipids, phosphocholine from glycerophosphocholine, identification of glutamate, glutathione, and 2-hydroxyglutarate, etc	Quantitative mapping of proteins/peptides and metabolites present within the brain tumors with high sensitivity and spatial resolution

## Author Contributions

All authors listed have made a substantial, direct, and intellectual contribution to the work and approved it for publication.

## Funding

This work was supported by funding obtained from University Research Foundation (URF), Perelman School of Medicine at the University of Pennsylvania, Philadelphia, USA (PI: SC, PhD, DABMP), extramural research grants [(BT/RLF/Re-entry/16/2015), and (CRG/2021/000668)] from Department of Biotechnology (DBT) and Science & Technology (DST), Govt. of India, respectively (PI: MK).

## Conflict of Interest

JB was employed by Median Technologies. The remaining authors declare that the research was conducted in the absence of any commercial or financial relationships that could be construed as a potential conflict of interest.

## Publisher's Note

All claims expressed in this article are solely those of the authors and do not necessarily represent those of their affiliated organizations, or those of the publisher, the editors and the reviewers. Any product that may be evaluated in this article, or claim that may be made by its manufacturer, is not guaranteed or endorsed by the publisher.
